# Ameliorative effect of liprotide-encapsulated vitamin D3 on blood glucose, calcium homeostasis, and vitamin D level in a vitamin D and calcium deficient rat model

**DOI:** 10.3389/fnut.2025.1514179

**Published:** 2025-01-28

**Authors:** Gemala Anjani, Reza Achmad Maulana, Sylvia Rahmi Putri, Faizah Fulyani, Ahmad Syauqy, Diana Nur Afifah, Fitriyono Ayustaningwarno, Refani Alycia Kusuma, Zulfatul Masruroh

**Affiliations:** ^1^Department of Nutrition Science, Faculty of Medicine, Diponegoro University, Semarang, Indonesia; ^2^Department of Nutrition Science, Faculty of Public Health, Ahmad Dahlan University, Yogyakarta, Indonesia; ^3^Department of Medical Biology and Biochemistry, Faculty of Medicine, Diponegoro University, Semarang, Indonesia

**Keywords:** encapsulation, blood glucose, calcium, liprotide, vitamin D3

## Abstract

Vitamin D3, recognized for its higher bioavailability and direct cell utilization, plays a vital role in the human body. Applying *β*-lactoglobulin with oleic acid (β-Lg-AO) as an encapsulating agent is anticipated to protect and enhance the transport of vitamin D3 to the gastrointestinal system. This study aimed to evaluate effect of liprotide-encapsulated vitamin D3 in a vitamin D-deficient rat on blood glucose, Vitamin D and calcium status. This is pre-post intervention. 24 mice were divided into 4 groups: (K-) normal rats; (K+) rat model of vitamin D and calcium deficiency; (P1) rat model of vitamin D and calcium deficiency with vitamin D_3_ intervention; (P2) rat model of vitamin D and calcium deficiency with liprotide-encapsulated vitamin D_3_ intervention. The administered dose of vitamin D3 was 180 IU (2 mL solution). Liprotide-encapsulated vitamin D3 intervention in vitamin D and calcium deficiency rats can significantly increase vitamin D (25 (OH)D) and calcium levels (*p* < 0.05). The increase in vitamin D (25 (OH)D) level was 53.69 ng/mL, and the increased calcium level was 4.38 mg/dL. Blood glucose levels of vitamin D-calcium deficiency rats decreased significantly (*p* < 0.05) by 39.87 mg/dL. Vitamin D3 encapsulated liprotide improves vitamin D and calcium in the blood more effectively than vitamin D3 without encapsulation in deficient rats.

## 1 Introduction

Vitamin D is a fat-soluble vitamin that is essential for the human body. The incidence of vitamin D deficiency is significant, with recent research indicating a prevalence rate of 5.9% in the United States, 7.4% in Canada, and 40% in Europe (1). In Indonesia, 45.1% of children aged 1 to 18 years (2) and 82% of women of reproductive age have vitamin D deficiency (3). Several factors contribute to vitamin D deficiency, including dietary intake and lifestyle (3). Most foods contain minimal amounts of vitamin D, with only a few food groups providing substantial quantities of vitamin D (4). In Indonesia, dietary consumption of vitamin D sources remains low, and the common practice in Southeast Asia of avoiding sun exposure also contributes to the high rate of vitamin D deficiency (3). Adequate exposure to UVB radiation from sunlight is essential for vitamin D production in the skin, as it converts 7-dehydrocholesterol into vitamin D3 (5, 6). The study by Chalcraft et al. (5) shows that exposure to sunlight significantly increases serum D3 levels in both younger and older people.

Vitamin D functions as a transcription factor that modulates gene regulation, including genes CYP24A1 (cytochrome P450 family 24 subfamily A member 1) and CAMP (cathelicidin antimicrobial peptide), exerting physiological effects on the body (1, 7). Absorption of vitamin D3 with high bioavailability can significantly increase 25-hydroxyvitamin D (25 (OH)D) levels in the bloodstream, providing a reliable marker for an individual’s vitamin D status. Calcifediol (25 (OH)D) is the most stable form of vitamin D, allowing for accurate and reliable measurement (8). This form of vitamin D is hydrolyzed in the liver and then moves through the bloodstream to the kidneys, which is further hydroxylated by the enzyme 1α-hydroxylase (CYP27B1). This enzyme is abundant and possesses excellent hydroxylation capacity (9). A hydroxyl group (-OH) is added to 25 (OH) D, converting it into 1,25-dihydroxy vitamin D (1,25 (OH)2D) or calcitriol, the active form of vitamin D utilized by the body. In circulation, 1,25 (OH)2D binds to the vitamin D-binding protein (DBP), which functions as a transporter, facilitating its delivery to target organs that possess vitamin D receptors (VDRs). The active form of vitamin D binds to VDRs located on the nuclear membranes of cells within various tissues and organs (10). VDRs are key mediators of vitamin D’s cellular mechanisms in the body (1, 9), with over 60 cell types and more than 200 genes identified as targets for VDR activation (11).

Vitamin D has a role in maintaining glucose tolerance (12). It stimulates insulin receptor expression, enhances the insulin response to glucose, and regulates membrane calcium flux to ensure sufficient intracellular cytosolic calcium for insulin secretion, thereby helping to reduce insulin resistance (13). Vitamin D deficiency can indirectly impact calcium levels during insulin secretion, affecting signal transduction and glucose transporter activity. Insulin secretion is a calcium-dependent process in which vitamin D indirectly supports pancreatic *β* cell function by regulating calcium flow and extracellular calcium levels. Consequently, vitamin D deficiency can destabilize intracellular and extracellular calcium, which in turn affects normal insulin secretion (14).

Vitamin D3 can be sourced from animal products, including fish oil, meat, and egg yolks. In this instance, vitamin D3 is already active and available for the body’s utilization. Alternatively, vitamin D3 is synthesized in human skin as provitamin D3 (7-dehydrocholesterol) is converted into cholecalciferol (vitamin D3) through exposure to UV-B radiation from sunlight (15, 16). This vitamin is characterized by its insolubility in water, instability under acidic conditions, and susceptibility to oxidation (17). The absorption efficiency of conventional vitamin D3 is around 50% (18). The maximum plasma concentration (Cmax) of cholecalciferol varies significantly from 0.58 ng/mL to 3,040 ng/mL, but the liver’s maximum concentration of vitamin D3 ranges from 0.67 ng/mL to 3480.9 ng/mL. The time to reach the maximum plasma concentration (Tmax) of cholecalciferol was 15.28 h and terminal half-life (T1/2) ranges from 1.21 h for 1,23S,25-trihydroxyvitamin D3 to 7.98 h for cholecalciferol (19). Encapsulation protects vitamin D3 from oxidation and acidic pH conditions in the gastrointestinal tract (20).

The combination of protein and fat forms a liprotide system, capable of encapsulating specific compounds, substances, or molecules. The liprotide structure consists of a core of fatty acids surrounded by a partially denatured protein layer (21). This protein layer enhances the solubility of fatty acids, enabling liprotides to effectively transport hydrophobic molecules within a hydrophilic environment. Additionally, the protein coating facilitates the uptake and delivery of fatty acids to target cells or hydrophobic surfaces. The fatty acid portion of the liprotide complex can form hydrophobic interactions with various hydrophobic compounds, substances, or molecules, such as vitamin D3 (22). The study by Frislev et al. (23) show that liprotides are effective tools of delivering cholesterol to cells and membranes. Various studies have been conducted to improve the stability of vitamin D, including utilizing milk proteins, specifically *α*-lactalbumin and *β*-lactoglobulin, for vitamin D binding (22).

*β*-lactoglobulins are proteins employed to deliver vitamin D3, docosahexaenoic acid, and genistein (24) and as components of whey proteins, they hold potential as carriers for vitamin D3 (25, 26).β-lactoglobulin has been demonstrated to be a very complex protein molecule that can undergo a variety of pH-induced transitions, thiol-disulfide turnover, and dimer-monomer conversion. It can also populate a variety of transitory states, including liquid globules and other types of aggregates (27). *β*-lactoglobulin (BLG) has two key advantages over other food proteins such as it resists pepsin digestion due to its abundance of charged amino acids, rigid beta-sheet structures, and disulfide bonds, and it is slowly digested by trypsin in the small intestine. These properties make BLG an effective encapsulant for controlled release of sensitive compounds. Additionally, BLG’s natural ligand-binding capacity makes it an excellent carrier for nutraceuticals (26). The role of the protein is to enhance the solubility of fatty acids, which makes liprotides suitable for transporting hydrophobic molecules in hydrophilic environments. Specifically, *β*-lactoglobulin combined with oleic acid forms a liprotide system that can be effectively used for vitamin encapsulation (28). Oleic acid is one of the fatty acids utilized in producing liprotides due to its abundant availability, ease of acquisition, effectiveness, and lower cytotoxicity than other cis fatty acids. Due to its low solubility, the handling and preparation of oleic acid are critical when formulating liprotide complexes. Oleic acid interacts with proteins in various ways, leading to variations in the oleic acid-to-protein ratio, which can influence these interactions (29). Previous study show that Tween 80/oleic acid composite vesicles showed excellent encapsulation for vitamin C and slow-release properties, highlighting their potential as antioxidant delivery systems in cosmetics (30).

Vitamin D3 will be encapsulated with liprotides composed of *β*-lactoglobulin and oleic acid (β-Lg-AO). Applying β-lactoglobulin with oleic acid (β-Lg-AO) as an encapsulating agent is anticipated to protect and enhance the transport of vitamin D3 to the gastrointestinal system. Therefore, this study will measure vitamin D3, calcium, and blood glucose levels in a vitamin D - calcium deficient rat model.

## 2 Materials and methods

### 2.1 Materials

Cholecalciferol vitamin D3 (≥98%, C9756), Ca^2+^ − depleted *α*-lactalbumin from bovine milk (≥85% pure), oleic acid, *β*-lactoglobulin, pepsin enzyme, sodium cholate, lipase enzyme, pancreatin enzyme, hydrochloric acid, sodium chloride, and potassium chloride were from Sigma-Aldrich.

### 2.2 Preparation of liprotide *β*-Lg-AO complexes

β-lactoglobulin at a 6 mg/mL concentration was mixed with 1.5 mg/mL oleic acid in 10 mM KOH (pH 10.5) and incubated for 39 min at 45°C. After incubation, the β-lactoglobulin sample was cooled and added with 50 mM Na_2_HPO_4_ and 150 mM NaCl. The pH was adjusted to 7.4 using HCl (22).

### 2.3 Preparation of liprotide-encapsulated Vitamin D3

Vitamin D3 was dissolved in 96% ethanol to a concentration of 115 mM and then further diluted in Milli-Q water. Vitamin D at a concentration of 280 μM was mixed with 4 mg/mL liprotide. The samples were homogenized using a vortex, centrifuged, and allowed to stand at room temperature (20–25°C) (22).

### 2.4 Ethical consideration

All studies were performed in accordance with globally recognized guidelines for the use and care of laboratory animals. All the experiments were started after approval of study protocol and ethical issues by Health Research Ethics Committee, Medical Faculty, Diponegoro University No 71/EC/H/FK-UNDIP/V11/2021.

### 2.5 Animal and treatments

This study involved 24 male Wistar rats, aged 8 weeks and weighing between 150 and 300 grams, which were divided into four groups: (1) Normal control group / K(−); (2) Vitamin D and calcium-deficient group/K(+); (3) Vitamin D and calcium-deficient group treated with vitamin D3/ P1; and (4) Vitamin D and calcium-deficient group treated with vitamin D3 encapsulated in *β*-Lg-AO/P2. The study protocol was structured as follows: Week 1 was dedicated to acclimatization, Weeks 2–3 were used to induce vitamin D and calcium deficiency, and Weeks 4–7 involved the intervention phase, and where rats received their respective treatments.

To establish a model of vitamin D and calcium deficiency, rats in the deficiency groups were initially fed a modified AIN-93 M diet for 2 weeks. After the deficiency induction phase, all groups were switched to a standard AIN-93 M diet for the remainder of the study. The intervention phase lasted 4 weeks and involved administering 1,000 IU/kg body weight (BW) of vitamin D3, equivalent to 180 IU for rats. The vitamin D3 was administered in two forms: 2 mL of non-encapsulated vitamin D3 (P1), and 2 mL of vitamin D3 encapsulated in *β*-Lg-AO (P2). The solvent for liprotide encapsulation was water, while virgin coconut oil (VCO) was used for the non-encapsulated vitamin D3 formulation. Rats received their respective treatments through sonde administration (31).

### 2.6 Assessment of serum biochemistry parameters

Data collection on vitamin D, calcium, and blood glucose levels was conducted twice: pre-and post-intervention. Blood samples were collected via the retro-orbital plexus (31). Blood vitamin D levels were measured using an Enzyme-linked Immunosorbent Assay (ELISA) kit (ABclonal 25OHVD ELISA-kit) (31). Blood calcium levels were assessed using Atomic Absorption Spectroscopy (AAS) (32), while fasting blood glucose levels were measured using the Glucose Oxidase Peroxidase Aminoantipyrine (GOD-PAP) method (33).

### 2.7 Data analysis

Statistical analysis was conducted using SPSS software version 21 (IBM/SPSS Inc.). The research data were assessed for normality using the Shapiro–Wilk test. Paired T-test was applied to determine pre and post intervention differences in vitamin D3, calcium, and blood glucose levels. Differences in effects between groups were analyzed using One-Way ANOVA with *Post Hoc* follow-up tests. All results are presented as mean ± SD, and a *p*-value of <0.05 was considered statistically significant.

## 3 Results

### 3.1 Body weight and feed intake of rats during intervention

The feed intake of the rats was weighed daily during the study to monitor the amount of feed consumed by all groups of rats. During the vitamin D-calcium deficient-rats conditioning period, the K (+), P1, and P2 had lower feed intake than the K (−) group. During the intervention period, the K (−) group had higher feed intake than the P1 group and P2 group (Table 1).

**Table 1:** Feed intake of rats during the study.

Groups			AIN-93 M diet (g)			
	1st week	2nd week	1st week	2nd week	3rd week	4th week
K(−)	17.98	14.90	16.62	18.55	18.86	18.77

	Modified of AIN-93 M diet (g)		AIN-93 M diet (g)			
	1st week	2nd week	1st week	2nd week	3rd week	4th week
K(+)	19.21	17.33	16.55	17.81	17.71	18.00
P1	18.40	16.62	16.12	17.64	18.55	16.71
P2	18.17	15.21	14.79	16.40	17.93	17.13

**Figure 1:**
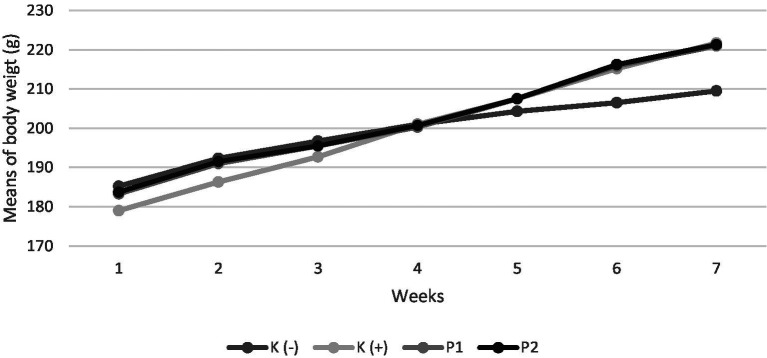
Body weight of rats during the study. Four groups of rats (*n* = 6 each group) consist of K(−): Normal control group, K+: vitamin D and calcium-deficient group, P1: Vitamin D and calcium-deficient group treated with vitamin D3 and P2: Vitamin D and calcium-deficient group treated with vitamin D3 encapsulated in *β*-Lg-AO.

Experimental animals were weighed every week during the study (Figure 1). At acclimatization, all groups had a body weight following the study’s inclusion criteria of 150–200 g. After the conditioning period of Vitamin D-Calcium deficiency for 14 days, all groups experienced an increase in body weight. After the intervention period, each group showed an increase in body weight.

### 3.2 Vitamin D levels in blood

**Figure 2:**
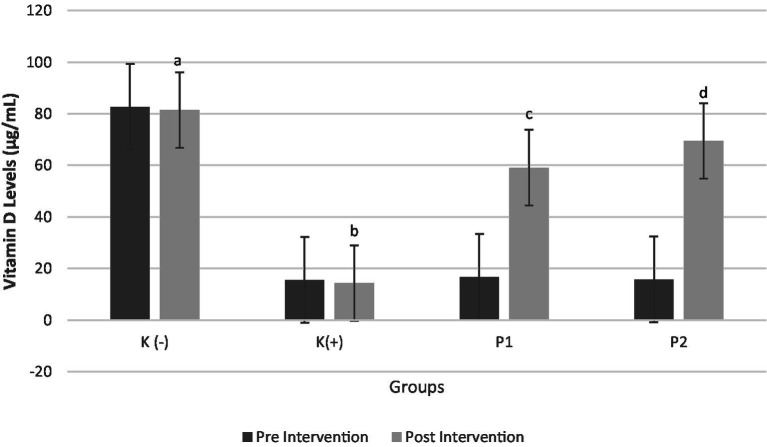
Vitamin D3 Levels in Blood Pre and Post Intervention Four groups of rats (n = 6 each group) consist of K(−): Normal control group, K+: vitamin D and calcium-deficient group, P1: Vitamin D and calcium-deficient group treated with vitamin D3 and P2: Vitamin D and calcium-deficient group treated with vitamin D3 encapsulated in β-Lg-AO. The error bars indicate the standard deviation from the mean. Numbers followed by superscript letters (a,b,c,d) differ to show significant differences (*p* < 0.05).

The changes in vitamin D levels before and after the intervention showed statistically significant differences (*p* < 0.05). Specifically, the administration of vitamin D3 (P1) and liprotide-encapsulated vitamin D3 (P2) led to a significant increase in blood vitamin D levels in rats initially deficient in vitamin D. The vitamin D levels in these rats increased by 253 and 340%, respectively, with the liprotide-encapsulated vitamin D3 (P2) exhibiting a significantly greater increase compared to the non-encapsulated vitamin D3 (P1). These results emphasize the superior efficacy of the encapsulated form in correcting vitamin D deficiency, with the observed differences being statistically significant (*p* < 0.05) as shown in Figure 2.

### 3.3 Calcium levels in blood

**Figure 3:**
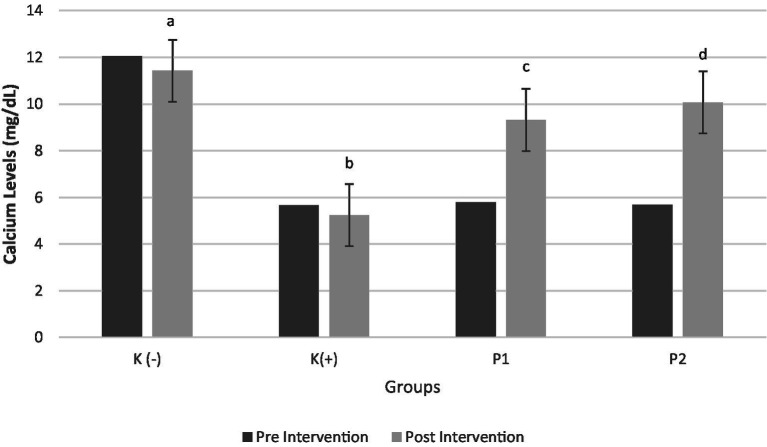
Calcium Levels in Blood Pre and Post Intervention Four groups of rats (n = 6 each group) consist of K(−): Normal control group, K+: vitamin D and calcium-deficient group, P1: Vitamin D and calcium-deficient group treated with vitamin D3 and P2: Vitamin D and calcium-deficient group treated with vitamin D3 encapsulated in β-Lg-AO. The error bars indicate the standard deviation from the mean. Numbers followed by superscript letters (a,b,c,d) differ to show significant differences (*p* < 0.05).

The study results indicated a positive correlation between calcium and vitamin D levels. Figure 3 presents significant differences in calcium levels before and after the intervention (*p* < 0.05). Rats were classified as calcium deficiency if blood calcium levels were < 6 mg/dL (34). The findings revealed that administration of vitamin D3 (P1) and liprotide-encapsulated vitamin D3 (P2) to calcium-deficient rats significantly increased blood calcium levels. Specifically, the blood calcium levels in rats treated with vitamin D3 (P1) increased by 60.79%, while rats treated with liprotide-encapsulated vitamin D3 (P2) showed an even greater increase of 76.9%, with both treatments yielding statistically significant results (*p* < 0.05). These results highlight the effectiveness of both vitamin D formulations in addressing calcium deficiency in the rats.

### 3.4 Blood glucose levels

**Figure 4:**
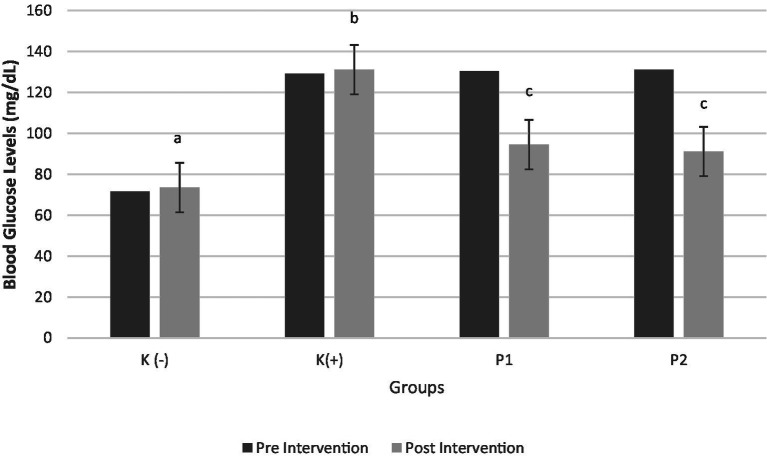
Blood Glucose Levels Pre and Post Intervention Four groups of rats (n = 6 each group) consist of K(−): Normal control group, K+: vitamin D and calcium-deficient group, P1: Vitamin D and calcium-deficient group treated with vitamin D3 and P2: Vitamin D and calcium-deficient group treated with vitamin D3 encapsulated in β-Lg-AO. The error bars indicate the standard deviation from the mean. Numbers followed by superscript letters (a,b,c,d) differ to show significant differences (*p* < 0.05).

Figure 4 shows significant differences in blood glucose levels before and after the intervention (*p* < 0.05). The results indicated that the deficient rats (K+) exhibited a significant increase in blood glucose levels (*p* < 0.05) compared to the normal rats (K-). The results of the study indicated that rats initially deficient in vitamin D exhibited a significant and marked increase in blood glucose levels compared to rats with normal vitamin D levels, with the observed difference being statistically significant (*p* < 0.05). In contrast, the administration of both vitamin D3 (P1) and liprotide-encapsulated vitamin D3 (P2) to the vitamin D-deficient rats resulted in a significant reduction in blood glucose levels (*p* = 0.001). Notably, no significant difference was found between the two treatments, suggesting that both vitamin D3 and its liprotide-encapsulated form had comparable effects in lowering blood glucose levels in the deficient rats. These findings imply that both forms of vitamin D may play an essential role in the regulation of blood glucose levels under conditions of vitamin D deficiency.

## 4 Discussion

Encapsulation technology provides a viable method for improving the stability and bioavailability of vitamin D3. This study showed that the intervention with liprotide-encapsulated vitamin D_3_ increased vitamin D3 in rats lacking in vitamin D and calcium. The significant increase in vitamin D levels observed in the P2 group suggests that liprotide-encapsulated vitamin D3 has higher bioavailability than non-encapsulated vitamin D3. Dalek et al. (35) showed that vitamin liposomes encapsulated vitamin D3 are absorbed more rapidly in the gastrointestinal tract. Their intrinsic stability enables efficient mixing with the aqueous phase and minimizes the impact of food components in the stomach. Additionally, the formation of smaller mixed micelles further accelerates the transfer of vitamin D3 across the mucous layer. Consequently, the absorption of cholecalciferol in liposomal formulations is expected to be significantly enhanced (35). Liprotides enhance the bioavailability of Vitamin D3 by forming nanostructures that encapsulate the hydrophobic vitamin, protecting it from degradation in the gastrointestinal environment, and facilitating its solubilization and absorption (22, 36). As demonstrated in studies by Jannik et al. (22), encapsulation of vitamin D in liprotides significantly enhances its stability, protecting the molecule from degradation by elevated temperatures, UV light, and oxidative processes.

Vitamin D levels in group P2 showed higher vitamin D levels and calcium levels than group P1. These results suggest that increased vitamin D levels in the blood are associated with elevated calcium levels. Furthermore, liprotide-encapsulated vitamin D3 (P2) was more effective in improving blood calcium levels compared to unencapsulated vitamin D3 (P1). Liprotide encapsulation enhances the bioavailability of vitamin D3, ensuring more effective absorption and sustained metabolic effects (37). Vitamin D plays a crucial role in regulating the absorption of calcium and phosphorus, which are essential for bone mineralization, growth, and the maintenance of bone strength (38, 39). It activates the vitamin D receptor (VDR), a gene transcription factor involved in calcium homeostasis and metabolism (12). The active form of vitamin D (1,25 (OH)2D) stimulates the active transport of calcium across the intestinal wall. Specifically, 1,25 (OH)2D activates VDR in gastrointestinal epithelial cells, leading to the synthesis of calcium-binding proteins (CaBP-9 K) and the activation of calcium channels (TRPV6 and TRPV5), facilitating active calcium transport (40). Previous studies have consistently demonstrated a correlation (*r* = 0,203) between vitamin D3 levels and serum calcium, suggesting that vitamin D plays a crucial role in regulating calcium homeostasis (41).

Moreover, liprotide-encapsulated vitamin D3 offers an advantage in modulating glucose metabolism. The encapsulation process protects vitamin D3 from rapid degradation, allowing for prolonged and more efficient action. Form of vitamin D3 enhances insulin sensitivity, stimulates insulin receptor activity, and promotes better glucose tolerance by improving insulin secretion in response to blood glucose levels (37). A previous study showed that high-dose vitamin D supplementation improved glucose homeostasis in infertile men, as evidenced by lower fasting serum insulin concentrations and HOMA-IR (42). In this study vitamin D3 (P1) and liprotide-encapsulated vitamin D3 (P2) in deficient rats significantly reduced blood glucose levels (*p* = 0.001), with no notable difference between the two treatments. Many previous studies have emphasized the role of vitamin D in various metabolic processes occurring in *β*-pancreatic cells within the Langerhans islets. The role of pancreatic beta cells in producing the hormone insulin as a regulator of blood glucose in circulation can be influenced by vitamin D levels (43). In cases of vitamin D deficiency, there is an increased risk of developing diabetes (44). Vitamin D deficiency has been specifically associated with reduced insulin secretion, insulin resistance, and the development of type 2 diabetes mellitus. Moreover, β-pancreatic cells possess specific receptors for vitamin D that play a crucial role in regulating insulin secretion. Vitamin D can stimulate insulin receptor activity, initiate insulin responses to glucose, and ensure adequate intracellular calcium levels for insulin secretion by modulating cell membrane calcium fluxes. Thus, vitamin D has a beneficial impact on insulin resistance (45, 46). Furthermore, vitamin D intake has been shown to influence insulin resistance positively and correlates with insulin secretion in patients with type 2 diabetes mellitus. Increased serum vitamin D concentrations have a favorable effect on insulin homeostasis (47). Previous studies showed that vitamin D supplementation, particularly at moderate to high doses (≥1,000 IU/day), is associated with a significant reduction in the incidence of type 2 diabetes (T2DM), especially in individuals with prediabetes. This suggests that adequate vitamin D levels may play a role in preventing the progression to diabetes in those at higher risk (48).

In summary, vitamin D3 and liprotide-encapsulated vitamin D3 have a significant effect on key physiological parameters in vitamin D and calcium-deficient rats. Both forms of vitamin D significantly increased blood vitamin D levels, with liprotide-encapsulated vitamin D3 showing a greater increase than non-encapsulated vitamin D3. Additionally, both treatments effectively improved blood calcium levels, with liprotide-encapsulated vitamin D3 showing a more pronounced effect. Furthermore, vitamin D3 and liprotide-encapsulated vitamin D3 significantly reduced blood glucose levels in the vitamin D-deficient rats, with no notable difference observed between the two treatments. These findings suggest that both forms of vitamin D play a crucial role in regulating calcium and glucose metabolism, making liprotide-encapsulated vitamin D3 a promising approach for improving vitamin D status and related metabolic functions.

## Data Availability

The original contributions presented in the study are included in the article/supplementary material, further inquiries can be directed to the corresponding author.
